# Vasoplegia treatments: the past, the present, and the future

**DOI:** 10.1186/s13054-018-1967-3

**Published:** 2018-02-27

**Authors:** Bruno Levy, Caroline Fritz, Elsa Tahon, Audrey Jacquot, Thomas Auchet, Antoine Kimmoun

**Affiliations:** 1 CHRU Nancy, Service de Réanimation Médicale Brabois, Pôle Cardiovasculaire et Réanimation Médicale, Hôpital Brabois, Vandoeuvre les Nancy, France; 2 INSERM U 1116, Groupe Choc, Equipe 2, Faculté de Médecine, Vandoeuvre les Nancy, France; 3 Université de Lorraine, Faculté de Médecine, Nancy, France

**Keywords:** Vasoplegic syndrome, Circulatory failure, Catecholamines, Septic shock, Vasoconstrictor agents

## Abstract

Vasoplegia is a ubiquitous phenomenon in all advanced shock states, including septic, cardiogenic, hemorrhagic, and anaphylactic shock. Its pathophysiology is complex, involving various mechanisms in vascular smooth muscle cells such as G protein-coupled receptor desensitization (adrenoceptors, vasopressin 1 receptors, angiotensin type 1 receptors), alteration of second messenger pathways, critical illness-related corticosteroid insufficiency, and increased production of nitric oxide. This review, based on a critical appraisal of the literature, discusses the main current treatments and future approaches. Our improved understanding of these mechanisms is progressively changing our therapeutic approach to vasoplegia from a standardized to a personalized multimodal treatment with the prescription of several vasopressors. While norepinephrine is confirmed as first line therapy for the treatment of vasoplegia, the latest Surviving Sepsis Campaign guidelines also consider that the best therapeutic management of vascular hyporesponsiveness to vasopressors could be a combination of multiple vasopressors, including norepinephrine and early prescription of vasopressin. This new approach is seemingly justified by the need to limit adrenoceptor desensitization as well as sympathetic overactivation given its subsequent deleterious impacts on hemodynamics and inflammation. Finally, based on new pathophysiological data, two potential drugs, selepressin and angiotensin II, are currently being evaluated.

## Background

### Definition(s) of vasoplegia

Known as “vasodilatory shock”, this condition includes multiple and diverse etiologies (e.g., septic, cardiogenic, neurogenic, and anaphylactic shock) and ultimately results in uncontrolled vasodilation, otherwise termed “vasoplegia”. The pathophysiology of vasoplegia is multifactorial and includes activation of several intrinsic vasodilatory pathways and a vascular hyporesponsiveness to vasopressors [1]. Vasoplegia occurring post-surgery is called postoperative vasoplegic syndrome or vasoplegic syndrome. In clinical practice, vasoplegia can be assessed clinically by the vasopressor dosage necessary to maintain mean arterial blood pressure (MAP) and by the drop in diastolic blood pressure reflecting vasoplegia [2]. Invariably, the necessity to use a high-dose vasopressor is highly indicative of vasoplegia, especially in the case of normal cardiac function. For further details, the reader is invited to consult the pathophysiological article published in the same series.

However, vascular responsiveness to vasopressors is probably better suited than vasoplegia for characterizing the state of vessels during shock. While the term vasoplagia refers to the static diameter of the vessel in response to specific intra-luminal and transmural pressures, vascular responsiveness to vasopressors refers to the dynamic response of the vessel to endogenous and/or exogenous vasoconstrictor agents [1].

The present review was written based on a critical and personal appraisal of the literature. It focuses only on treatment-based pathophysiology of vasoplegia and the benefits or drawbacks of each associated therapeutic option for all types of shock, irrespective of their origin. Nevertheless, there is a clear lack of data with regard to vasoplegia treatments in non-septic shock.

## Vasoplegia occurs in all shock states

Although initially ascribed to septic shock, it is now apparent that the majority of mechanisms explaining or associated with vascular hyporesponsiveness to vasopressors (inflammation, nitric oxide (NO), potassium and calcium channels, adrenomedullin, and free radicals) are also common to hemorrhagic shock, cardiogenic shock (including in post-cardiopulmonary bypass patients), anaphylactic shock, and, more generally, during ischemia-reperfusion, such as cardiac arrest or multiple trauma [3–5].

## A treatment-based pathophysiological approach to vascular hyporesponsiveness to vasopressors

**Fig. 1:**
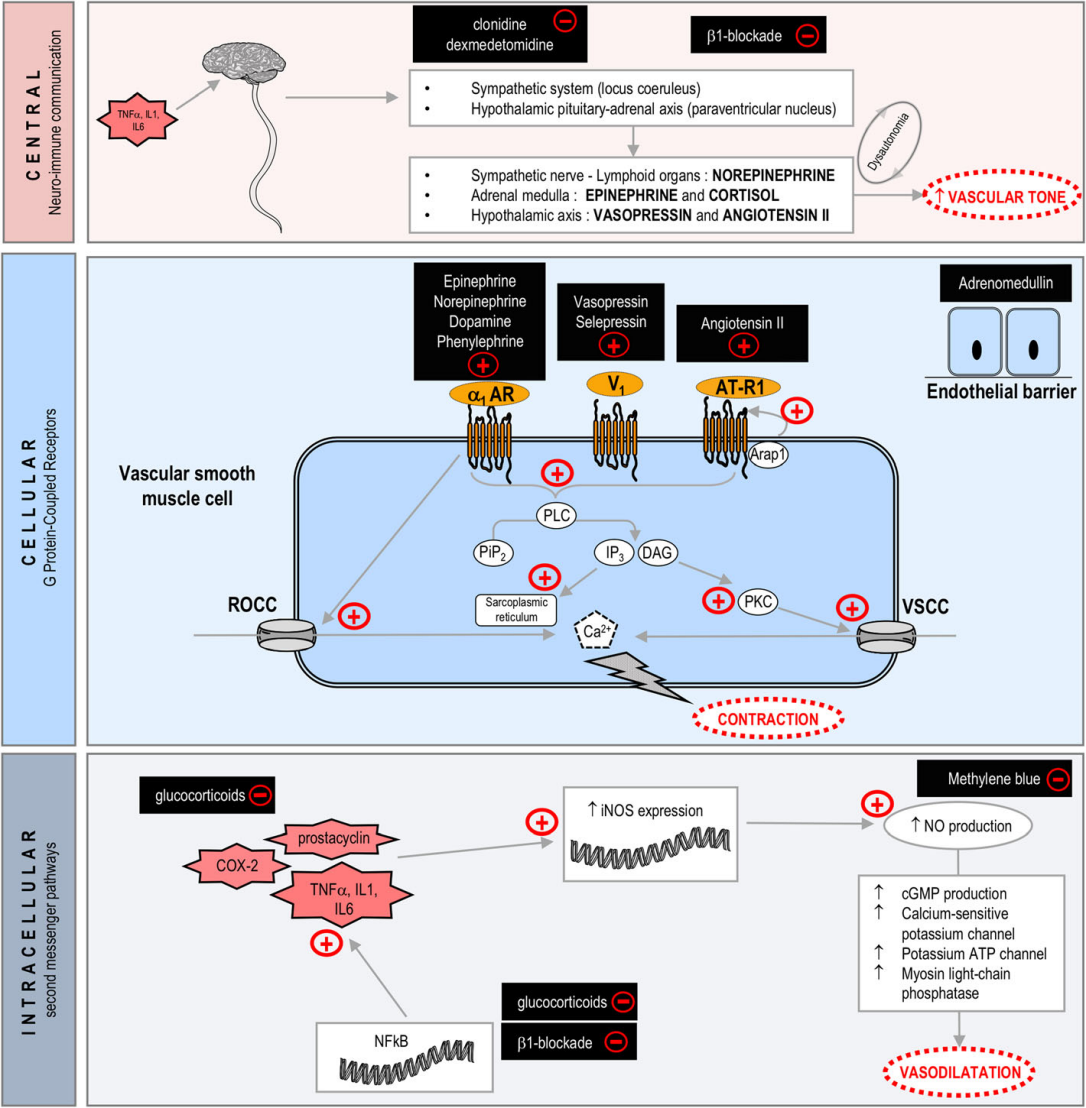
The principal mechanisms involved in the regulation of vascular tone during vasoplegia as well as treatment options at the central, cellular, and intracellular levels. *Central level.* Inflammatory triggers such as tumor necrosis factor α (TNF, interleukin (IL)-1 and IL-6 activate the neuro-immune system. This activation leads to norepinephrine, epinephrine, cortisol, vasopressin, and indirectly angiotensin II production in order to counteract vasoplegia. Overactivation of this system may be treated at this integrative level with α_2_ agonists and selective β_1_ blockers. *Cellular level.* G-protein-coupled receptors are predominantly involved in vascular smooth muscle cell contraction: α_1_ adrenoceptors (*α*_*1*_*AR*), vasopressin 1 receptors (V1R), and angiotensin type 1 receptors (*AT-R1*). These receptors activate phospholipase C (*PLC*) with generation of inositol 1,4,5 trisphosphate (*IP3*) and diacylglycerol (*DAG*) from phosphatidyl inositol 4,5 bisphosphate (*PiP*_*2*_). DAG stimulates protein kinase C (*PKC*), which in turn activates voltage-sensitive calcium channels, while IP3 activates sarcoplasmic reticulum calcium channels. α_1_ARs increase intracellular calcium by receptor-operated calcium channels (*ROCC*) stimulation. Available treatments at this level are epinephrine, norepinephrine, dopamine, phenylephrine, selepressin, vasopressin (*V1*), and angiotensin II. Adrenomedullin primarily acts on endothelial cells. *Intracellular level.* Translocation of nuclear factor-κB (NF*-*κB) into the nucleus induces pro-inflammatory cytokine production. These cytokines enhance inducible nitric oxide synthase (*iNOS*) expression and overproduction of NO. This molecule activates cyclic guanosine monophosphate production as a mediator of vasodilation. Available treatments at this level are glucocorticoids (at different steps), β_1_ blockade, and methylene blue. Vascular sensitive calcium channel (*VSCC*)

Here, we limit our description to the pathophysiological mechanisms involved in vascular hyporesponsiveness to vasopressors where treatments are currently available or soon will be. Thus, certain crucial mechanisms have been omitted, although they are described elsewhere in this series of articles on vasoplegia. The following three levels will be described: central (neuro-immune communication), cellular (G protein-coupled receptors (GPCRs)), and intracellular (alteration of second messenger pathways) (Fig. 1).

### Neuro-immune communication

Shock states are primarily associated with a concomitant initial activation of the sympathetic system in the locus coeruleus and the hypothalamic pituitary-adrenal axis in the paraventricular nucleus by stimulation of baro- and chemoreceptors and inflammatory cytokines such as tumor necrosis factor (TNF)α, interleukin (IL)-1 and IL-6. These two systems are both co-activated such that activation of one also tends to activate the other. Consequences include the release of norepinephrine from sympathetic nerve extremities in lymphoid organs, epinephrine from the adrenal medulla, and cortisol from the adrenal cortex. Of note, vasopressin release is also under the control of baro- and chemoreceptors characterizing the autonomic system [6]. Moreover, vasopressin also increases the activation of the hypothalamic pituitary-adrenal axis [7]. Finally, vasopressin and angiotensin II interact synergistically at a peripheral level in vascular smooth muscle in order to increase calcium concentrations [8]. Together, all of these systems participate in the maintenance of vascular responsiveness, particularly during the initial stage of shock state.

Sustained activation of the sympathetic system is associated with dysautonomia, a syndrome characterized by loss of cardiovascular variability with inappropriate tachycardia, excessively elevated catecholamine levels with concomitant adrenoceptor desensitization, and pro-inflammatory states leading to poor outcome [9]. This triad participates in vascular hyporesponsiveness to vasopressors during shock states.

### G-protein-coupled receptors

The three major receptors (adrenergic, vasopressin 1 (V1), and angiotensin type 1 (AT1) receptors) involved in the regulation of vascular tone are GPCRs. During shock states, adrenergic, V1, and AT1 receptors undergo similar desensitization processes. Sustained agonist activation such as in the initial phase of shock is associated with phosphorylation of GPCRs by GPCR kinases (GRKs). This process appears to be activated early, even following transient agonist stimulation, and is a major cause of vascular hyporesponsiveness to the three major vasopressors. The decreasing affinity of α adrenergic receptors for various molecules such as endotoxin is known to enhance desensitization [10]. AT1 receptors are downregulated within the first hours after experimental septic shock. This process is associated with low blood pressure and low systemic vascular resistance [11]. However, others have also demonstrated that AT1 receptors are primarily downregulated, although not by their agonist but rather through deficient expression of the AT1 receptor-associated protein Arap1. Arap1 is known to enhance the transport of the AT1 receptor from endosomes to the plasma membrane [12]. Finally, V1 receptors appear to be less sensitive to agonistic stimulation due to low circulating concentrations of vasopressin in blood even during shock states [7]. After an initial increase in concentration at shock onset, a decrease in vasopressin plasma levels is most often observed [13].

### Alteration of second messenger pathways

In addition to the desensitization process, other mechanisms are also highly involved in vascular hyporesponsiveness to vasopressors. For instance, expression of inducible nitric oxide synthase (iNOS) is enhanced during shock states in vascular smooth muscle cells (VSMCs) while NO production is increased a thousand-fold. Endotoxin and proinflammatory cytokines increase iNOS expression and NO production [14]. NO activates cyclic guanosine monophosphate (cGMP) production as well as calcium-sensitive potassium channels, potassium ATP channels, and myosin light chain phosphatase, all of which contribute to vasodilation [15]. Other mechanisms equally involved in vasodilatation include prostacyclin and cyclooxygenase 2 (COX2) pathways, although with no currently known positive therapeutic consequences [16].

Critical illness-related corticosteroid insufficiency (CIRCI), which occurs in 50 % of septic shock patients, has a major impact on vascular hyporesponsiveness to vasopressors [17]. Involved mechanisms include insufficient synthesis of cortisol, tissue resistance to cortisol, and an excessive proinflammatory response. Injuries are observed at all levels of the hypothalamo-hypopituitary axis. Adrenocorticotropic hormone (ACTH) secretion may be impaired by shock-induced anatomical lesions of the pituitary axis [18]. It has also long been known that adrenal necrosis and/or hemorrhage may be due to shock state and particularly septic shock [18]. Tissue resistance has multifactorial causes involving, among others, downregulation of glucocorticoid receptor α at the tissue level and reduction of cortisol delivery to septic locations. Excessive proinflammatory secretion also impacts ACTH secretion. Thus, TNFα and IL-1, massively released during septic shock, downregulate ACTH and cortisol production.

Consequences of CIRCI on hemodynamic parameters during shock states are extensive with vascular hyporesponsiveness to phenylephrine and low blood pressure. Underlying mechanisms involve disinhibition of NF-κB with upregulation of iNOS responsible for NO over-production.

## Vasoplegia treatment

### The use of adrenergic vasopressors

Vascular hyporeactivity-associated hypotension is clearly associated, both significantly and independently, with mortality [19]. After volume resuscitation, the use of catecholamines is considered to be the cornerstone of septic shock hemodynamic treatment [20]. This therapeutic class includes dopamine, epinephrine, norepinephrine, and phenylephrine. All of these molecules increase MAP by stimulating the α_1_ adrenergic receptor. Nevertheless, aside from phenylephrine, all of the above catecholamines stimulate other adrenergic receptors, leading to various hemodynamic, metabolic, and inflammatory effects [21, 22]. Comparison of the affinity of these different drugs for receptor subtypes as well as the effects associated with receptor stimulation is depicted in Table 1. Hence, the choice of best adrenergic vasopressor should take into account not only its vasopressor effect but also its cardiac, metabolic, microcirculatory, and immune effects.

**Table 1:** Adrenoceptors and vasoporessin and angiotensin receptors: subtypes, cellular mechanisms, vascular effects, and main localization

Receptor type	Adrenoceptor α		Adrenoceptor β			Vasopressin receptor			Angiotensin receptor	
	α_1_	α_2_	β_1_	β_2_	β_3_	V1a	V1b	V2	AT-R1	AT-R2
Drug affinity	Ep = NEp	Ep > NEp	Ep < NEp	Ep > NEp	Ep = NEp	Vasopressin	Vasopressin	Vasopressin	Angiotensin II	
						Selipressin		Desmopressin		
						Terlipressin		Terlipressin		
Vascular agonistic effects	Vasoconstriction	Vasorelaxation	Vasorelaxation	Vasorelaxation	Unknown	Vasoconstriction	NA	Vasorelaxation	Vasoconstriction	Vasorelaxation
Inflammation effect	Down-regulation ++	Down-regulation ++	Down-regulation +	Down-regulation +	Unknown	Down-regulation	Down-regulation	Down-regulation	Unknown	Unknown
Second messengers	PLC-DAG-IP3-PKC	AC-cAMP-PKA (−)	AC-cAMP-PKA (+)		Unknown	PLC-DAG-IP3-PKC	PLC-DAG-IP3-PKC	AC-cAMP	PLC-DAG-IP3-PKC	Unknown
Main localization	Post-synaptic receptors	Extrajunctional post-synaptic receptors	Post-synaptic receptors	Extrajunctional post-synaptic receptors	Unknown	Vascular smooth muscle cells	Anterior pituitary gland	Vascular smooth muscle cells	Medullary interstitial cells +++	Adrenal gland
	Vascular smooth muscle cells α_1_>>α_2_		Vascular smooth muscle cells β_2_>>β_1_			Hepatocytes		Basolateral membrane of collecting duct	Brain	Brain
	Coronary circulation (large artery)		Coronary circulation (large and small arteries) β_1_>>β_2_			Myometrium		Vascular endothelium	Myocytes	Myocytes
	Cerebral circulation (not smaller vessels)		Cerebral circulation			Platelets			Vascular smooth muscle cells	
	Skeletal and pulmonary (α>>β)		Skeletal and pulmonary						Renal glomeruli and proximal tubules	
			Splanchnic	Splanchnic (β_2_>>β_1_)						

Inspired from [21]. Plus signs indicate stimulation; minus signs indicate inhibition; equal signs indicate similar drug affinity

*AC* adenylate cyclase, *cAMP* cyclic adenosine monophosphate, *DAG* diacylglycerol, *Ep* Epinephrine, *IP3* inositol 1,4,5 trisphosphate, *NA* not applicable, *NEp* Norepinephrine, *PKA* protein kinase A, *PKC* protein kinase C, *PLC* phospholipase C

### The current recommendations

A recent Cochrane analysis concluded that there was not sufficient evidence to prove that any one vasopressor was superior to others in terms of mortality and that the choice of a specific vasopressor may, therefore, be individualized and left to the discretion of treating physicians [23]. Despite low levels of evidence, the Surviving Sepsis Campaign (SSC) published several recommendations based on the physiological effects of vasopressors and selection of inotrope/vasopressor combinations in septic shock outlined in an extensive number of literature reviews [20].

### Norepinephrine as a first line-agent

Norepinephrine is a very potent and reliable vasopressor. It increases MAP without any concomitant increase in heart rate. Generally, cardiac index is increased due to both a rise in end-diastolic stroke volume through a mobilization of splanchnic unstressed volume and to a direct effect on cardiac myocytes due to β_1_ adrenergic receptor stimulation [24]. Norepinephrine has numerous advantages when compared to other vasopressors, including: a) a very potent vasopressor effect equivalent to epinephrine and phenylephrine and higher than dopamine [25]; b) contrary to epinephrine, norepinephrine does not act on β_2_ adrenergic receptors—hence, lactate levels do not increase and may be used to guide resuscitation [26]; c) contrary to dopamine and epinephrine, norepinephrine increases cardiac index without increasing heart rate and thus without excessively increasing myocardial oxygen consumption [27]; d) contrary to phenylephrine, which acts only on α_1_ adrenergic receptors, norepinephrine also acts on cardiac β_1_ adrenergic receptors and may therefore preserve ventricular–arterial coupling [28].

Finally, adrenergic vasopressors have potential side effects such as increased oxidative stress, interaction with cellular energy metabolism, and/or modulation of the inflammatory response [22]. As a result, a new concept has emerged called “decatecholaminization”, which consists in using non-catecholamine vasopressors in order to decrease catecholamine exposure [29].

### Vasopressin as a second line agent or a catecholamine-sparing agent

Patients with severe septic shock often require very high doses of norepinephrine in order to achieve the target MAP, thereby potentially leading to adverse side effects [30]. The SSC suggests adding either vasopressin (up to 0.03 U/min; weak recommendation, moderate quality of evidence) to norepinephrine with the intent of raising MAP to target, or adding vasopressin (up to 0.03 U/min; weak recommendation, moderate quality of evidence) to decrease norepinephrine dosage. The rationale for vasopressin use is that there is a relative vasopressin deficiency in septic shock such that addition of exogenous vasopressin restores vascular tone by acting on non-adrenergic receptors, increases blood pressure, thereby reducing norepinephrine requirements, and possibly has favorable effects on cytokine production [31–33]. Globally, vasopressin is as effective as norepinephrine in increasing MAP and, when used in combination with norepinephrine, low vasopressin doses have a norepinephrine-sparing effect. The VASST study, in which vasopressin was used in substitutive doses (< 0.04 U/min), showed no overall improvement in mortality [34]. In a post-hoc analysis, however, patients with less severe septic shock (i.e., < 15 μg.min^−1^ of norepinephrine) at vasopressin initiation had a lower 28-day mortality rate compared with norepinephrine-only infusion (26.5 vs 35.7 %; *p* = 0.05). Higher doses of vasopressin have been associated with cardiac, digital, and splanchnic ischemia and should be reserved for situations in which alternative vasopressors have failed [35]. The VANCS trial compared norepinephrine to vasopressin in treating vasoplegia syndrome after cardiac surgery [36]. The primary endpoint was a composite of mortality or severe complications (stroke, requirement for mechanical ventilation for longer than 48 h, deep sternal wound infection, reoperation, or acute renal failure) within 30 days. The primary outcome occurred in 32 % of vasopressin patients compared to 49 % of norepinephrine patients (unadjusted hazard ratio 0.55; 95 % CI 0.38 to 0.80; *p* = 0.0014). With regard to adverse events, the authors found a lower occurrence of atrial fibrillation in the vasopressin group (63.8 vs 82.1 %; *p* = 0.0004) and no difference between groups with regard to rates of digital ischemia, mesenteric ischemia, hyponatremia, or myocardial infarction. These results thus suggest that vasopressin can be used as a first-line vasopressor agent in postcardiac surgery vasoplegic shock and improves clinical outcomes. Lastly, the VANISH study, assessing vasopressin versus norepinephrine with or without adding hydrocortisone (factorial 2X2 study) as initial therapy in septic shock, demonstrated no improvement in the number of kidney failure-free days [37]. Addition of hydrocortisone as an adjunct in the two vasopressor groups was used to upregulate receptor expression on VSMCs and to enhance anti-inflammatory effects.

Terlipressin, a long acting vasopressin analog with predominant V1 receptor activity, has also been proposed. When compared to norepinephrine, terlipressin significantly reduced catecholamine requirements, and led to fewer rebound hypotension events, without increasing bilirubin levels [38]. There is still ongoing debate regarding its ideal dose and mode of administration (continuous infusion despite long half-life or intermittent administration). Notwithstanding, terlipressin may result in pulmonary vasoconstriction and affect coagulation systems whereas vasopressin does not [38]. Hence, terlipressin is not considered to offer a greater advantage compared to vasopressin due to its longer half-life and clinical evidence that supports its use in circulatory shock remains scarce [20]. In spite of these caveats, the place of terlipressin is currently being evaluated in two ongoing studies (NCT03038503 and NCT02468063).

### Phenylephrine use should be limited

Phenylephrine is a pure α_1_ adrenergic agonist for which clinical trial data are limited. It has the potential to produce splanchnic vasoconstriction. Moreover, in a model of rat septic shock, phenylephrine use has been associated with a detrimental effect on intrinsic cardiac function [39]. Lastly, among patients with septic shock in US hospitals affected by the 2011 norepinephrine shortage, Vail et al. [40] found that the most commonly administered alternative vasopressor was phenylephrine. Patients admitted to these hospitals during times of shortage had higher in-hospital mortality.

### A critical view of the recommendations

Two recommendations should be addressed. The first recommendation concerns the use of epinephrine as a second-line agent and the second regards the use of dopamine in highly selected patients. The relevance of using epinephrine in association with norepinephrine should be discussed since a) epinephrine markedly increases lactate levels and may therefore preclude the use of lactate clearance to guide resuscitation [25], b) norepinephrine and epinephrine both act on α_1_ adrenergic receptors so there is no therapeutic value in adding the same molecule type when norepinephrine has failed to increase MAP, and c) the combination of norepinephrine and dobutamine, allowing the separate titration of vasopressor and inotropic effects, is more logical than using epinephrine alone. Therefore, we firmly believe that epinephrine has no place in septic shock treatment with the exception of countries with limited resources (it is cheaper than norepinephrine). In these countries, it is acceptable to use epinephrine since no data support a difference in efficacy, mortality, or morbidity [29, 41]. With regard to dopamine, there is currently ample evidence that norepinephrine or epinephrine is more efficient in restoring MAP and that both drugs could be used through a peripheral venous access [42, 43]. Thus, dopamine should no longer be used in septic shock. Moreover, in a randomized study comparing dopamine and epinephrine in the treatment of shock, a subgroup analysis of 280 patients with cardiogenic shock showed dopamine to be associated with an increase in 28-day mortality compared to norepinephrine [27].

## The future

### Selepressin, an improved vasopressin receptor agonist?

Since vasopressin comparably stimulates all vasopressin receptor subtypes (i.e., V1a, V1b, and V2 receptors), it may also have serious undesirable side effects through V2 stimulation (fluid accumulation, microvascular thrombosis, vasodilation) [44]. Selepressin, a short-acting selective V1a receptor agonist, may overcome these disadvantages [45]. Furthermore, selepressin does not induce release of the procoagulant Willebrand factor [46]. In a study by Maybauer et al*.* [47] describing the effects of selepressin in an ovine model of severe sepsis, the effects of V1a and V2 receptor activation were compared using selective V1a (selepressin) and V2 (desmopressin) receptor agonists. Accumulation of fluid was blunted by arginine vasopressin while reversed by selepressin. When selepressin was combined with desmopressin, fluid accumulation was restored to levels similar to the sepsis + vasopressin group. These findings were also confirmed by He et al. [48], who found that early administration of selepressin as first line vasopressor treatment improved MAP, cardiac index, blood lactate levels, lung edema, and fluid balance and was associated with higher survival rates compared to vasopressin and norepinephrine. In light of the above, several completed or currently ongoing clinical trials are investigating the clinical implications of selepressin. The preliminary results of two phase II trials (NCT01612676 and NCT01000649) showed that selepressin enabled the dose requirements of norepinephrine to be reduced. In addition, incremental doses of selepressin were found to reduce overall excessive fluid balance and were associated with higher rates of ventilator-free days, shock resolution, and patient survival within the first 7 days [49]. Accordingly, an ongoing double-blinded phase IIB/III, randomized clinical trial (NCT02508649) is studying the effects of selepressin compared to placebo on ventilator and vasopressor-free days.

### Angiotensin II

Activation of the renin–angiotensin–aldosterone system leads to angiotensin II production [50]. Angiotensin II acts by binding to specific GPCRs, namely AT1 and AT2 [51]. The main hemodynamic effects mediated by AT1 receptor activation include vasoconstriction, aldosterone secretion, vasopressin release, and cardiac remodeling [52]. In the ATHOS-3 study, patients with vasodilatory shock who were receiving more than 0.2 μg.kg^−1^.min^−1^ of norepinephrine or the equivalent dose of another vasopressor were assigned to receive infusions of either angiotensin II or placebo [53]. The primary end point was MAP response at 3 h after initiation of infusion, with response defined as an increase from baseline of at least 10 mmHg or an increase to at least 75 mmHg, without an increase in dose of background vasopressors. The primary endpoint was reached by more patients in the angiotensin II group than in the placebo group (*p* < 0.001). At 48 h, the mean improvement in the cardiovascular Sequential Organ Failure Assessment (SOFA) score was greater in the angiotensin II group than in the placebo group (*p* = 0.01). Serious adverse events were reported in 60.7 % of the patients in the angiotensin II group and in 67.1 % in the placebo group. Death by day 28 occurred in 75/163 patients (46 %) in the angiotensin II group and in 85/158 patients (54 %) in the placebo group (*p* = 0.12).

### Methylene blue

Inhibition of excessive production and activity of both NO and cGMP may be critical in the treatment of refractory vasodilatory shock occurring in cardiac bypass, septic shock, poisoning, and anaphylaxis patients. Methylene blue (MB) has several actions that may counteract the effect of increased NOS stimulation. First, it may antagonize endothelial NOS activity. Furthermore, it may scavenge NO directly and inhibit guanylate cyclase activity [54]. Experimental animal studies report that, in addition to a reduction in vasopressor requirements, inotropic support is reduced after the administration of MB, likely due to attenuation of ischemia/reperfusion injury [55]. In a human septic shock study, MAP and cardiac index were both found to be increased [56]. A systematic review of the literature regarding the use of MB in sepsis by Kwok and Howes [57] concluded that, while the studies were mostly observational, MB increased systemic vascular resistances and MAP; however, its effects on oxygen delivery and mortality are unknown. Moreover, all of the aforementioned studies are relatively old and likely do not take into account current recommendations.

The use of MB has been proposed not only for septic shock but also for treating vasoplegia after cardiac surgery, drug poisoning, anaphylactic shock, and post-reperfusion syndrome after liver transplantation [54]. Similar to septic shock, however, data are currently insufficient to propose MB as a first line agent [58].

The potential dangers of treatments targeting iNOS overexpression in septic shock should nonetheless be kept in mind. For example, non-selective iNOS blockers, while improving systemic vascular resistance and MAP, also reduce cardiac output and increase mortality in patients with septic shock [59]. Similarly, non-selective iNOS inhibition with tilarginine versus placebo in cardiogenic shock patients failed to reduce the mortality rate at 30 days [60]. Interestingly, there was also no difference in hemodynamic outcomes such as duration of shock. This negative result may be the consequence of the inhibition of other beneficial NO isoforms [61].

Despite these limitations, the place of MB in vasoplegia treatment is currently being evaluated in a number of ongoing studies (NCT03038503, NCT01797978, NCT03120637).

## Potential new strategies

### Very high doses of norepinephrine

Depending on the study, high doses of norepinephrine associated with excess mortality have been defined by a cut-off value ranging from 0.5 to 2 μg.kg^−1^.min^−1^, although converging evidence has recently confirmed the cut-off as 1 μg.kg^− 1^.min^− 1^ [30, 62]. Obviously, the level of MAP that is targeted should be taken into account.

Because these very high doses may be associated with potential deleterious effects, there is still controversy regarding increasing vasopressor dosage when conventional therapy fails to increase mean arterial pressure to the recommended target. The pharmacodynamic effects of catecholamines are characterized by a linear increase in effect, which is dependent on the logarithmic increase of the concentration, without any saturation at high doses [63]. Auchet et al. [62] found that a vasopressor dose higher than 0.75 μg.kg^− 1^.min^− 1^ was associated with a mortality of 86 % in patients with a SOFA score > 10 and with a mortality of 58 % in patients with a SOFA score < 10.

Moreover, the administration of high doses should be stopped in instances of serious adverse events. In two studies, myocardial, mesenteric, and digital ischemia occurred in less than 10 % of patients [62, 64]. Moreover, adding an additional vasopressor (vasopressin) in order to reduce norepinephrine dosage was not associated with a lower incidence of serious adverse events [64].

As a result, physicians should also consider an increase in norepinephrine dosage as a possible therapeutic option in instances of refractory hypotension associated with vasoplegia and adequate cardiac function, without the fear of ischemic complications.

## Modulation of the sympathetic system

### α_2_ Agonists

During a shock state, the inappropriate activation of the sympathetic system is associated with receptor desensitization [65]. One innovative approach may be to reduce sympathetic activity. α_2_ Agonists such as clonidine or dexmedetomidine (200 times more powerful than clonidine) act directly in the locus cœruleus. By binding to presynaptic α_2_ adrenergic receptors, these agonists also induce a negative feedback on norepinephrine secretion. Known pharmacological effects of this central down-regulation are hypotension, bradycardia, and sedation [66]. However, recent experimental studies in small and large animals have found that administration of α_2_ agonists, by reducing central sympathetic activity, also restores the response to vasoconstrictors such as α_1_ agonists or angiotensin II [67, 68]. One appealing hypothesis is that the reduction in sympathetic outflow allows a lesser desensitization of peripheral adrenergic receptors as well as a reduction in pro-inflammatory cytokine secretion. Direct vasoconstrictor effects of α_2_ agonists should also be taken into account.

### Selective β_1_ blockade

β_1_ Blockade seemingly restores vascular responsiveness to vasopressors. In 2013, Morelli et al. [69] demonstrated that esmolol, a selective β_1_ blocker, administered in hemodynamically stabilized septic shock patients, efficiently reduced heart rate without apparent side effects. Of greater interest, the authors observed a decrease in the dose of norepinephrine infused in the esmolol group compared to the placebo group. There are two prevailing hypotheses to explain this unexpected result on norepinephrine dose. First, in 2016, Morelli et al. [70] found that, in septic shock patients under esmolol, the decrease in heart rate was associated with improved arterial elastance, thus restoring ventricular–arterial coupling. Second, our team recently found that in experimental septic shock, esmolol infusion in rats, even at low doses that did not induce a reduction in heart rate, was associated with a better ex vivo vasoreactivity compared to non-treated animals. These beneficial effects appear to be associated with a downregulation of inflammatory pathways in vessels such as NF-κB [71].

Perspectives in adrenergic modulation could include both central reduction of sympathetic outflow by α_2_ agonists and peripheral downregulation of β_1_ adrenergic receptors by selective β_1_ blocker. Accordingly, Hernandez et al. [72] recently published an experimental study in which they compared the effects of dexmedetomidine and esmolol relative to lipopolysaccharide-control animals on exogenous lactate clearance in a sheep model of early endotoxic shock. The authors found that these two molecules were hemodynamically well tolerated and were associated with better exogenous lactate clearance. Correct dosages and hemodynamic tolerances of the combination of these two molecules nonetheless remain to be explored.

### Glucocorticoids

Many experimental studies have demonstrated that administration of glucocorticoids restores vascular responsiveness to vasopressors, likely through a non-genomic inhibition of the arachidonic acid cascade and a genomic inhibition of the nuclear translocation of the NF-κB transcription factor [73]. Moreover, glucocorticoids also inhibit the synthesis of iNOS and COX2 [74, 75]. Finally, low doses of glucocorticoids appear to restore vascular responsiveness to norepinephrine through an increase in α adrenergic receptor gene expression [76]. A clinical trial demonstrated that administration of low doses of hydrocortisone in septic shock patients tended to normalize the vascular responsiveness to phenylephrine [73]. However, results of a large clinical trial assessing the efficiency of low doses of hydrocortisone on mortality in septic shock patients yielded conflicting findings. Consequently, the SSC recommends against treating septic shock with low intravenous doses of hydrocortisone if hemodynamic fluids and catecholamines are able to restore stability. However, in the case of refractory septic shock, low doses of hydrocortisone (200 mg per day) may be administered [20]. Preliminary results of the APROCCHSS study (NCT00625209; involving hydrocortisone and fludrocortisone) revealed a beneficial effect on 90-day mortality and shock reversal.

### Vasopressor combinations

Depending on the efficacy/risk ratio, optimal vasopressor therapy could thus consist of a combination of agents acting on different receptors while minimizing doses of each agent and therefore possibly increasing overall safety. This paradigm was indirectly tested in both the VAAST and ATHOS-3 studies [34, 53], in which norepinephrine doses were decreased when adding vasopressin or angiotensin II. The combination allowed a decrease in total norepinephrine dose. Unfortunately, this decrease in dosage was not associated with a decrease in adverse events.

### Adrenomedullin blocking

Adrenomedullin is considered as a double-edge sword in septic shock. On the one hand, adrenomedullin supplementation improves endothelial barrier function, attenuates systemic inflammation, and reverses hypodynamic circulation and pulmonary hypertension in ovine endotoxemia. On the other hand, high levels of adrenomedullin are associated with short-term mortality and vasopressor requirement in both septic and cardiogenic shock [77, 78]. Finally, adrenomedullin binding has been found to blunt shock-related impairment in energy metabolism as well as to reduce nitrosative stress and attenuate systemic inflammatory response, all of which were ultimately associated with reduced kidney dysfunction and organ injury [79]. One ongoing study (NCT03085758) is currently comparing two doses of ADRECIZUMAB (a humanized murine monoclonal IgG1 antibody specifically binding the N-terminal region of human adrenomedullin) in patients with early septic shock and a high bio-adrenomedullin plasma concentration.

### A role for genomics and pharmacogenomics?

Pharmacogenomics could be applied to enhance efficacy and safety of drugs used for sepsis and septic shock, including norepinephrine, epinephrine, vasopressin, and corticosteroids, since known genomic variants intersect with these drugs. For example, Nakada et al. [80] demonstrated that β_2_ adrenergic receptor gene polymorphism was associated with altered responses to adrenergic agonists and mortality in septic shock. Nevertheless, the variant was only present in 5 to 7 % of the population, thereby rendering the elaboration of a specific test hazardous and likely very expensive.

## Conclusions

Vasoplegia is a common feature of all advanced shock states, with norepinephrine remaining the cornerstone of vasoplegia-induced hypotension. However, given our improved understanding of vasoplegia, management is likely to evolve from a standardized therapy with norepinephrine alone to a multimodal strategy with two or more vasopressors. Based on new pathophysiological data, numerous potential drugs are currently being investigated. Nevertheless, these new potential treatments or therapeutic strategies should be evaluated not only for their ability to increase arterial pressure but also for their capacity to improve survival or decrease major morbidity as well as for their effectiveness/cost ratio.

## Abbreviations

ACTH: Adrenocorticotropic hormone
AT1: Angiotensin type 1
AVP: Arginine vasopressin
cGMP: Cyclic guanosine monophosphate
CIRCI: Critical illness-related corticosteroid insufficiency
COX2: Cyclooxygenase 2
GPCR: G protein-coupled receptor
GRK: GPCR kinase
IL: Interleukin
iNOS: Inducible nitric oxide synthase
MAP: Mean arterial blood pressure
MB: Methylene blue
NO: Nitric oxide
SOFA: Sequential Organ Failure Assessment
SSC: Surviving Sepsis Campaign
TNF: Tumor necrosis factor
V1: Vasopressin type 1
VSMC: Vascular smooth muscle cell
